# The effect of finishing diet supplemented with methionine/lysine and methionine/**α**-tocopherol on performance, carcass traits and meat quality of Hanwoo steers

**DOI:** 10.5713/ajas.19.0209

**Published:** 2019-08-03

**Authors:** Farouq Heidar Barido, Dicky Tri Utama, Hae Song Jong, Juntae Kim, Chang Woo Lee, Yeon Soo Park, Sung Ki Lee

**Affiliations:** 1 Department of Applied Animal Science, College of Animal Life Sciences, Kangwon National University, Chuncheon 24341, Korea; 2 Faculty of Animal Science, Universitas Brawijaya, Malang 65145, Indonesia; 3 Gangwon Province Livestock Research Institute, Hoengseong 25266, Korea

**Keywords:** Hanwoo Steers, Methionine, Lysine, α-Tocopherol, Meat Quality

## Abstract

**Objective:**

This study aimed to compare the effects of diets with and without supplements of methionine/lysine (met/lys) and methionine/α-tocopherol (met/α-tocopherol) on the performance, carcass traits and meat quality of Hanwoo steers.

**Methods:**

A total of 21 Hanwoo steers were divided into three different groups. Each group consisted of 7 animals that received different dietary treatments for 120 days as follows: a control (C) diet composed of a basal diet with 74% total digestible nutrient and 12% crude protein; treatment 1 (T1) composed of a basal diet enriched with methionine 69%+lysine 31%; and treatment (T2) composed of a basal diet enriched with methionine 84.65%+α-tocopherol 15.35%. Daily supplementation was given using the top-dressing method (20 g/animal).

**Results:**

The met/lys groups yielded a *longissimus lumborum* with increased water holding capacity (p<0.01) and lower shear force value (p<0.05). Dietary met/lys did not have an adverse effect on the animal performance and carcass traits. Instead, these supplements contributed significantly to the higher protein content compared to the control diet (p<0.05). Myristic acid (C14:0) was the only fatty acid affected by the supplementation. While the met/α-tocopherol group led to significantly higher protein and oxymyoglobin contents during storage (p<0.05). It also contributed significantly to the lower shear force value and 2-thiobarbituric acid reactive substances score during storage (p<0.05) compared to the control and treatment 1 since the initial storage day. The met/α-tocopherol diet also yielded meat with a redder color (p<0.05) after 3 days of storage. However, it did not significantly contribute to the fatty acid profiles of Hanwoo steers.

**Conclusion:**

Met/lys supplementation resulted in higher protein scores, water holding capacity and lower shear force scores. While met/α-tocopherol supplementation contributes to the production of redder meat, reduces lipid oxidation, production of more tender meat and increases the content of protein and oxymyoglobin percentage.

## INTRODUCTION

Efforts have been applied to improve the production and meat quality of cattle. One of these efforts is through supplementation with limiting amino acids, alone or in combination with other nutrition, to meet the metabolizable protein requirements. This is not a new technological approach applied in livestock industries to achieve the optimum performance of the animal [1]. Moreover, this supplementation is proven to have an expected effect in non-ruminant animals. Methionine and lysine, as the first and second limiting amino acids, respectively, were reported to enhance the growth rate and carcass yield and slightly improve meat quality by decreasing the rate of protein denaturation and contributing to the tenderness of chicken breast meat [2]. Furthermore, methionine in the rumen-protected form is considered to be capable of supporting protein synthesis and inhibiting protein degradation through involvement in the synthesis of reduced glutathione (GSH), which naturally inhibits reactive oxidative species (ROS) [3]. Besides, supplementation with lysine increased the concentration of sarcoplasmic protein, which positively correlates with an increase in meat tenderness [4]. Studies have inferred that when methionine and lysine are absorbed efficiently in the ruminant’s small intestine, the animals retain more nitrogen and, consequently, the muscle fiber types and satellite cell populations and altered; these amino acids may serve as optimizing agents for growth rate and are critical for maximizing lean tissue deposition [5].

In addition, it is well recognized that lipid oxidation is one of the major factors leading to meat deterioration, lowering the quality of meat by the production of off-flavors, reduction of polyunsaturated fatty acids (PUFAs), and consequently lower consumer preferability due to the unfresh color of the meat and possibly the formation of toxic compounds such as peroxides and aldehydes [6]. Therefore, vitamin E (α-tocopherol) is suggested as a dietary supplement, considering its ability to improve meat quality and inhibit lipid oxidation of muscle by increasing the concentration of α-tocopherol within the meat, which can reduce the existence of ROS [7]. There is still a limited amount of information related to the effect of mixing amino acids (methionine), either with lysine or with tocopherol, on the growth performance, carcass traits and meat quality of cattle, especially Hanwoo steers, during the fattening period. Therefore, this study aimed to investigate the effect of diets enriched with amino acid complexes and antioxidants on the growth performance, carcass quality and meat quality of Hanwoo steers.

## MATERIALS AND METHODS

### Animal care

The protocol for animal handling in this study was approved by the Institutional Animal Care and Use Committee (IACUC) at the Gangwon Province Livestock Research Institute.

### Animals, diets and feeding

This study utilized twenty-one Hanwoo steers with an initial weight of 712.57±17.26 kg. These animals were then randomly assigned into three different pens. Each pen consisted of 7 animals. The formulation of the diet given was based on the animal’s body weight to meet their nutritional requirements. It was formulated based on the guidelines given by NRC [8] for the finishing stage. The first group was fed a basal diet consisting of a concentrate and rice straw that contained 74% total digestible nutrient and 12% crude protein (CP) as a control (C). Two other groups were fed a basal diet (74% TDN and 12% CP) supplemented with either mixture 1 (methionine 69% [13.89 g]+lysine 31% [6.29 g], methionine/lysine [met/lys]) as treatment 1 (T1) or mixture 2 (methionine 84.65% [16.93 g] +α-tocopherol 15.35% [3.07 g], methionine/α-tocopherol [met/α-tocopherol]) as treatment 2 (T2). Rumen protected methionine, lysine (NOW Foods, Seoul, Korea) and α-tocopherol (Life Extension Official, Fort lauderdale, FL, USA) was supplemented in the form of powder in order to complete recommended guidelines by NRC [8]. Daily supplementation was given using the top dressing method (20 g/animal). Animals were fed for 120 days at Gangwon Provincial Livestock Research Institute’s farm.

### Finishing performance

Individual body weight was recorded at the beginning and after 120 days of feeding. Total weight gain and average daily gain (ADG) were calculated.

### Carcass traits and sampling

After 120 days of the finishing period, animals were slaughtered to measure the effects of the treatment diets on carcass traits. Carcass weight, yield grade (hot carcass weight, back fat thickness and ribeye area) and quality grade (marbling score, meat color, fat color, firmness and maturity) were evaluated based on the Korean carcass grading system. The strip loin (*longissimus lumborum*) of each animal was cut and packed in polyethylene zipper bags at 24 h postmortem and subsequently transported to the laboratory. Samples were stored in a chilling room at 2°C±2°C overnight. The strip loin from one animal was sliced into six steaks (2 cm thick). To observe the storage effect on color, pH, lipid oxidation and freshness, three steaks from each animal were put on styrofoam, wrapped with oxygen-permeable film and stored at 5°C±0.5°C in the dark for 3, 6, and 9 days. The other two were used for cooking loss and shear force analyses, while the remainder was used for the analyses of color, pH, lipid oxidation and freshness at day 0 of storage. The analysis of proximate composition, water-holding capacity (WHC) and fatty acid composition were also performed using day 0 samples. Analysis of day 0 samples was performed on day 2 postmortem. Samples used for fatty acid analysis were stored at −24°C under vacuum and thawed overnight prior to analysis.

### Proximate composition

Sample was ground using food grinder (HMF-1600 PB, Hanil Electric, Seoul, Souh Korea) with medium speed for 10 s. Proximate composition was determined according to Association of Official Analytical Chemists (AOAC) method [9]. The content of sample moisture was done by drying the samples within the oven at 105°C for 24 h. While for the content of crude fat was conducted according to the ether extraction by Soxhlet system. Nitrogen content was observed by performing assesment with the Kjeltec system (2200 Kjeltec Auto Distillation Unit, Foss, Hilleroed, Sweden) and CP was calculated as nitrogen content multiplied by 6.25. Crude ash was determined by burning the samples in the muffle furnace at 550°C for 12 h.

### Instrumental surface color

The meat color was measured right after blooming phase was completely achieved on its surface (1 h after slicing). Colorimeter (CR-400 Konica Minolta Sensing Inc., Tokyo, Japan) was used to record sample’s Lightness (L*), redness (a*), and yellowness (b*). The source of illuminant light C (2° observer) 8 mm aperture was calibrated using a white plate (Y = 93.6, X = 0.3134, y = 0.3194). Measurement was performed 5 times for each sample.

### Water-holding capacity, cooking loss, and shear force

The WHC was measured based on method by NRC [10] with slight modification. Shortly explained, 5 gram of ground samples were transfered into centrifuge tubes, sealed and then subjected for heating in water bath for 30 min with temperature of 75°C. The tube was then cooled in flowing cold water for 30 min, it was subsequently centrifuged at 980 *g* for 10 min at the temperature of 24°C (1248R, Labogene, Lynge, Hovedstaden, Denmark). The supernatant was then decanted and measured. Moisture content of both raw and the supernatant was determined according to AOAC method [9].

Cooking loss is defined as weight loss after subjected to heating until medium degree of doneness was achieved (68°C to 72°C internal temperature). Briefly, samples were weighed to get initial weight (W1) of 70±5 g. Samples in duplicate were then placed in the polyethylene zipper bags and cooked by immersing in water bath with temperature of 80°C. Cooked samples were subsequently discarded from the bags, cooled down at 2°C±2°C for 30 min and weighed (W2). The percentage of cooking loss was obtained by calculating weight loss (W1W2) against W1.

Samples from cooking loss was subsequently used to measure tenderness (toughness) of the meat by performing Warner-Bratzler Shear Force test using TA-XT2*i* Plus (Stable Micro Systems, Surrey, UK). The method was according to [11] where the sample was made into 1.5 cm×1 cm size, it was then well placed under the V blade, cut through the strand with a constant speed through the gap of insturment’s table (assay parameters were: pre-test speed: 2.0 mm/s; test speed: 1.0 mm/s; post-test speed: 10 mm/s). Each sample was repeated for eight times.

### pH and 2-thiobarbituric acid reactive substances

Method to obtain the pH score was according to method by Utama et al [11] where 5 g of sample was added with 45 mL of distilled water and subsequently mixed with a homogenizer (PH91, SMT Co., Ltd., Chiba, Japan) at 10,000 rpm for 60 second. The pH score of the well homogenized meat was documented using a pH meter. Calibration of pH meter was done by using technical buffer solutions consisted of acid (pH 4.01), alkali (pH 9.00) and neutral (pH 7.00) with an automatic temperature compensation program (Seven Easy pH, Mettler-Toledo GmbH, Greifensee, Switzerland).

The measurement of lipid oxidation was performed using the 2-thiobarbituric acid reactive substances (TBARS). Sample in amount of 0.5 grams in 25-mL TBARS test tube was prepared in three repetitions, 0.1 g of antioxidant mixture (consisting of 54% propylene glycol, 40% tween 20, 3% butylated hydroxytoluene, and 3% butylated hydroxyanisole) was transfered into the tube, subsequently 3 mL of 1% thiobarbituric acid in 0.3% NaOH was added into the mixture. Right after vortexing, 17 mL of 2.5% trichloroacetic acid in 36 mM HCl was added and the tube was closed. The sample was subjected for heating in a water bath (BW-20G, Biotechnical Services, Inc., North Little Rock, AR, USA) with temperature of 100°C for 30 min. The tube was then immersed in cold water for another 15 min. Aqueous sample in amount of 5 mL was transfered into a new 15 mL centrifuge tube and mixed with 3 mL of chloroform. The mixture was then subjected for centrifugation with the condition of 2,400×g for 30 min at 4°C (1248R, Labogene, Lynge, Denmark) to separate from the dirt. The absorbance was measured at 532 nm by using uv-spectrophotometer (UV-mini 1240 PC, Shimadzu Corp., Kyoto, Japan) against blank (distilled water was used to replace sample). Each sample was repeated three times. Data was expressed in the form of mg malondialdehyde/kg sample.

### Myoglobin content

One gram of beef sample was homogenized with 8 mL sodium phosphate solution (0.04 mol/L, pH 7). It was subsequently centrifuged at 10,000 g for 15 minutes. Supernatant formed was filtered through a Whatman filter paper. Extract absorbance was read at 572, 565, 545, and 525 nm against a blank using a spectrophotometer (iMark680, Bio-Rad, Hercules, CA, USA). The proportion of the DMb, OMb, and MMb was calculated by using a formula.

### Fatty acid composition

The determination of fatty acid composition was performed using an Agilent gas chromatography system (6890N, Agilent Technologies, Santa Clara, CA, USA) with an auto sampler (7683, Agilent Technologies, USA). Meat fat was extracted according to [12] with a chloroform-methanol (2:1 v/v) solution and prepared in duplicate. This method as described by [9] converted fatty acids into methyl esters. Converted fatty acid was subsequently dissolved in 1.5 mL of hexane. A total of 1 μL sample was then injected into the gas chromatography port by the auto sampler. The setting of injector was: 250°C with a split ratio of 100:1. Separation of fatty acid methyl esters was performed using a WCOT-fused silica capillary column (100 m×0.25 mm i.d., 0.20 μm film thickness; Varian Inc., Palo Alto, CA, USA) with a 1.0 mL/min helium flow. While setting of the oven was: 150°C/1 min, 150°C to 200°C at 7°C/min, 200°C/5 min, 200°C to 250°C at 5°C/min, and 250°C/10 min. The temperature of the detector was 275°C. Identification of fatty acid was obtained by comparing each sample’s peak with the retention time of fatty acid standards (47015-U, Sigma-Aldrich Corp., LLC., St. Louis, MO, USA). Data of peak area of each identified fatty acid can be used to obtain the proportion (%) of each against total identified peak area.

### Statistical analysis

The determination of significances for treatment groups (pH, TBARS, color, and myoglobin) during storage days was by using linear mixed model of two-way analysis of variance (ANOVA). While to determine the significant different for animal performance, carcass traits, proximate content, WHC, cooking loss, shear force, and fatty acid composition were compared with those of control sample by performing one-way ANOVA. Analyses were conducted using R version 3.5.1 with the “Agricolae” library (The R Foundation for Statistical Computing, Vienna, Austria).

## RESULTS

### Finishing performance

Table 1 displays the effect of ruminally protected met/lys and met/α-tocopherol supplementation on the finishing performance of Hanwoo steers. No differences were found (p>0.05) for final weight between the control and all treatments. However, a higher numerical score was found for cattle treated with met/α-tocopherol, with differences ranging from 11.00 kg to 17.43 kg, compared to control and treatment with met/lys, respectively. The same findings were also found for weight gain and ADG. The weight gain of the control, treatment 1, and treatment 2 groups was 67.14, 72.29, and 83.29 kg, respectively. The ADG of the control, treatment 1, and treatment 2 groups was 0.56, 0.60, and 0.69 kg, respectively.

### Carcass traits

The effects of dietary supplements of met/lys or met/α-tocopherol are shown in Table 2. No differences were found (p> 0.05) for all observed traits. However, numerically, cattle treated with a diet supplemented with met/α-tocopherol possessed better scores for hot carcass weight, ribeye area, backfat thickness, meat color, firmness, and quality grade. It is estimated that met/α-tocopherol treatment resulted in 39 kg and 38 kg of higher carcass weight compared to met/lys treatment and the control, respectively. On the other hand, supplementation with met/lys gave better results for the numerical marbling score.

### Meat quality

Feeding cattle with diets supplemented with either met/lys or met/α-tocopherol yielded meat with higher protein content (p<0.01), as shown in Table 3. However, there were no differences in protein content between either met/lys or met/α-tocopherol treatment. In addition, the moisture, fat and ash contents did not differ between the control and the treatments.

Cattle from both treatment groups responded positively to the diet by producing meat with a significantly lower shear force value (p<0.05), which indicates that both treatments produced meat with more tender characteristics. However, there were no significant differences between treatment diets. For the WHC score shown in Table 2, only supplementation with met/lys significantly affected (p<0.01) the WHC of the meat. However, the percentage of cooking loss did not differ significantly among samples with 29.45%, 27.64%, and 28.65% for control, treatment 1 and treatment 2, respectively.

The storage experiment was carried out for 9 days in a chilling room at 2°C±2°C. The rate of meat lipid oxidation was recorded through TBARS analysis which shown in Table 4, results was expressed as mg malondialdehyde/kg meat. A significant increase in the TBARS value (p<0.001) was observed during storage for all samples. However, cattle treated with treatment 2 had the most stable TBARS value throughout storage followed by treatment 1 and the control. Treatment 2 showed a significantly lower TBARS value (p<0.05) compared to the control and treatment 1 since the initial day of storage and this was retained until day 9. Treatment 1 resulted in better storage stability only at day 9 of storage (p<0.05) compared to the control. Analysis of variance did not indicate an interaction between storage and treatment (p>0.05).

The effect of met/lys and met/α-tocopherol supplementation on meat pH during storage is reported in Table 4. The pH score was inconsistent. During the initial storage and day 6 of storage, meat from cattle treated with met/α-tocopherol supplementation had significantly lower pH scores compared to the meat from the control and treatment 1 steers. On days 3 and 9, the pH content did not differ between the control and treatments. The pH score for all samples showed a significant increase (p<0.001) from day 0 to day 9 of storage. The highest pH score was observed from the control, followed by treatment 1 and treatment 2 with 5.61, 5.55, and 5.49, respectively.

The Commission Internationale de l’Eclairage (CIE) value changes were recorded in Table 5. The CIE lightness value (L*) of all samples did not change significantly during storage days (p>0.05). The difference was only observed on the final storage day (p<0.05), with the highest score found for meat from cattle treated with met/α-tocopherol followed by the control diet and treatment with met/lys. In contrast, the CIE redness value (L*), which indicates discoloration of the meat, did differ among all samples during storage (p<0.001). However, meat from cattle treated with met/α-tocopherol diet retained redness stability better than meat from those treated with T1 or the control diet (p<0.001) on day 3 and it showed superior scores on day 9 of storage. The redness of the meat did not differ between the control and treatment 1 diets on any storage day (p>0.05). Instrumental color changes for yellowness were observed during storage for all samples (p<0.001). The most significant decrease was observed for the control samples followed by treatment 1 and treatment 2 samples. However, there were no significant differences (p>0.05) at any interval of storage except at day 9. Treatment 2 had the highest score for yellowness at day 9, followed by treatment 1 and the control. However, there were no significant differences found between treatment 1 and treatment 2 on any storage day.

The formation of oxymyoglobin, metmyoglobin and deoxymyoglobin is shown in Table 5. Changes in oxymyoglobin were observed in all samples during the storage period (p< 0.001). Significant differences were documented between the control and both treatments at days 6 and 9 of storage, where both treatment diets resulted in higher oxymyoglobin percentages compared to the control diet (p<0.05). However, the oxymyoglobin level between the met/lys and met/α-tocopherol treatments did not differ on any storage day (p>0.05). Numerically, meat from cattle treated with the diet supplemented with met/α-tocopherol had a higher percentage of oxymyoglobin compared to the control and met/lys treatments. In contrast, the level of metmyoglobin increased significantly during storage (p<0.001) in all samples. Numerically but not statistically, the formation of metmyoglobin was highest in the control, followed by treatment 1 and then treatment 2 samples. Similarly, the percentage of deoxymyoglobin increased significantly with each interval of storage (p<0.01), with no significant differences found between the control diet and both treatment diets (p>0.05).

### Fatty acid composition

Diet supplemented with either met/lys or met/α-tocopherol had a limited effect on the intramuscular fatty acid of Hanwoo beef which can be seen in Table 6. The significant difference (p<0.05) was solely found in the C14:0 (myristic acid) content; cattle treated with a diet supplemented with met/lys produced meat with the highest percentage of C14:0, followed by cattle treated with met/α-tocopherol supplementation and the control treatment. Saturated fatty acid levels did not differ among any of the samples. Similarly, monounsaturated fatty acid (MUFA) and polyunsaturated fatty acid (PUFA) levels did not differ statistically among treatments. Additionally, the percentages of n3, n6 and n3/n6 were also not significantly different among treatments (p>0.05). Among all identified fatty acids, the major contributor to MUFA was oleic acid (C18:1n9), followed by palmitoleic acid (C16:1n7). Among PUFA, the major constituents were linoleic acid (C18:2n6), alpha-linoleic acid (C18:3n3), arachidonic acid (C20:4n6) and eicosapentaenoic acid (C20:5n3).

## DISCUSSION

Hanwoo steers were assessed for their performance after 120 days of feeding with either met/lys or met/α-tocopherol supplementation. Significant differences were not observed for final weight, weight gain or ADG of cattle either treated with diets supplemented with met/lys and met/α-tocopherol. This is in agreement with explained by Park et al and Prado et al [13,14], where a positive growth response from ruminally protected met/lys supplementation only occurred during the growth period, and no positive response was found in the finishing period. While there was also no effect on performance could be observed from the cattle treated with diet supplemented with α-tocopherol [15].

It was expected that treatment could enhance carcass traits, as occurs in nonruminant animals. Theoretically, methionine and lysine are two limiting amino acids that cannot be sufficiently provided by animals with a low protein diet [16], and the inclusion of these limiting amino acids could augment growth efficiency [14]. However, the data obtained in this study on carcass traits displayed no differences in all observed parameters (p>0.05). The same trend was shown even with high levels of dietary supplementation of met/lys by Prado et al [14]. High supplementation with met/lys had a limited effect on the carcass traits of Friesian steers. Additionally, the response to supplementation with met/lys showed an inconsistent pattern [17]. Dietary supplementation with met/α-tocopherol also showed a limited effect on carcass characteristics. It solely contributed to the higher yield of hot carcass weight and a higher numerical quality grade. In line with the present study, a study by Prado et al and Lee et al [14,18] also found that dietary supplementation with a combination of sulfur and vitamin E had a limited effect on the longissimus muscle of Hanwoo bulls.

Differences were obtained for the protein content of *longissimus lumborum* muscles of Hanwoo steers fed either treatment compared to the control diet. Dietary supplementation with either met/lys or met/α-tocopherol seemed to promote more protein synthesis. The mechanism relates to its role in the initiation of translation of protein synthesis [19]. Supported by the results of Min et al [6], supplementation with rumen protected methionine may lead to increased anabolic metabolism of proteins, which subsequently regulates the accretion of proteins. Moreover, methionine-based supplementation is considered to result in protein oxidation protection of the meat by promoting a combination of reduced GSH enzymes and animal stress reduction [21]. However, met/lys or met/α-tocopherol supplementation did not significantly contribute to the augmentation of crude fat and moisture content of the meat.

The water content of the meat is an essential quality parameter to be considered by the meat processor. Due to its relation to the final yield of the processed meat, it has economic implications. However, it is also essential in terms of eating [22]. Meat tenderness directly correlates with the acceptability of meat by consumers; thus, it is an important factor that should be considered in terms of meat quality [23]. The present study found that dietary met/lys significantly improved meat quality by decreasing the shear force value and increasing the WHC. It is inferred that supplementation with lysine plays a critical role in the mechanism of WHC augmentation, as occurs in poultry meat [24]. Lysine supplementation increases the concentration of sarcoplasmic protein and the ratio of soluble sarcoplasmic: myofibrillar protein, which can be used as an indicator of muscle alterations. As a result of muscle fiber type and satellite cell population alterations from type I fiber which is small and oxidative to type IIX fiber type which is larger, more stable to oxidation, glycolytic fiber types, and has ability to retain more water [5].

All samples showed an initial pH score below 5.6, which indicates that animals were not in a stressful condition when slaughtered. It also indicates that the meat was not pale soft exudative meat [25]. Treatment 2 resulted in a significantly lower pH score (p<0.05) compared to the control and treatment 1 at the beginning of storage, and this lower score was retained at day 6 of storage. A significant increase in the TBARS value (p<0.001) was observed during storage for all samples. Meat from cattle the fed treatment 2 diet had the most stable TBARS value throughout storage, followed by meat from cattle fed treatment 1 and the control diets. Treatment 2 resulted in a significantly lower TBARS value (p<0.05) compared to the control and treatment 1 since the initial day of storage, and this trend was retained until day 9. Treatment 1 resulted in better storage stability only at day 9 of storage (p<0.05) compared to the control. Analysis of variance did not indicate an interaction between storage and treatment (p>0.05).

Myoglobin reflects the freshness of the meat and is an important marker for meat discoloration, which correlates with the rate of lipid oxidation [18]. The myoglobin score directly corresponds to the color of the meat. A higher score of oxymyoglobin is more desirable for consumers due to the brighter red color formed and there is definite bias against tan or brown discoloration caused by the formation of metmyoglobin [3]. The present study documented significant differences between the control and both treatments at days 6 and 9 of storage, where both treatment diets resulted in higher oxymyoglobin percentages compared to the control diet (p<0.05). The coloration of red color and changes in oxymyoglobin values are strongly correlated with the rate of lipid oxidation [18]. This is because lipid oxidation could enhance myoglobin oxidation through the reactivity of primary and secondary products derived from unsaturated fatty acids. These results indicate that both treatments could effectively inhibit the rate of myoglobin oxidation by promoting antioxidant agents, as explained by Lee et al [21]. Methionine could promote the GSH enzyme, which naturally develops to inhibit ROS. However, supplementation with met/α-tocopherol could extend the color of the meat by inhibiting the rate of lipid oxidation and retarding the formation of metmyoglobin [7].

Nine fatty acids were identified within the beef samples. Dietary supplementation with both met/lys and met/α-tocopherol had a limited effect on the intramuscular fatty acid profile of Hanwoo beef. The content of PUFAs did not differ across samples. This finding agrees with that of the previous study by Parr et al [26] that although supplemented met/lys is well absorbed and protected from ruminal degradation, it did not significantly affect the PUFA level. Moreover, all groups of cattle received the same type of basal diet consisting of rice straw and a concentrate. However, cattle treated with a diet supplemented with met/α-tocopherol tended to have a numerically higher percentage of linoleic acid (C18:2n6), alpha-linoleic acid (C18:3n3), and arachidonic acid (C20:4n6) compared to those treated with the control and treatment 1. In addition, a significant difference was solely found (p< 0.05) in myristic acid (C14:0), where cattle treated with met/lys had the highest percentage. Similar to the present result, Strasia et al [27] also reported that a sufficient supply of well-protected limiting amino acids positively increased the fatty acid profile of Hanwoo steers. An n6/n3 over 4 is recommended [28,29]. This study found an n6/n3 average of 9.00, 10.10, and 9.71 for the control, treatment 1, and treatment 2, respectively, which is included in the normal range.

## CONCLUSION

In conclusion, supplementation of both met/lys and met/α-tocopherol during fattening to Hanwoo steers had no significant effect on growth performance and carcass weight and yield grade. Supplementation of met/lys resulted in a higher score of protein content, WHC and a tendency of lower shear force value and amount of cooking loss compared to control diet, which is economically profitable due to more augmentation of protein, and less product shrinkage. Therefore, supplementation with met/lys could be beneficial for Hanwoo steers during the fattening program. While met/α-tocopherol supplementation contributes to the production of redder meat, reduces lipid oxidation, production of more tender meat and enhances the protein and oxymyoglobin content, which is preferred by the consumer. However, further study to determine the optimum ratio of supplementation is needed to achieve the best effects on the desirable traits of Hanwoo meat.

## Figures and Tables

**Table 1:** Body weight gain of Hanwoo steers fed with different supplementary methionine/lysine and methionine/α-tocopherol

Variable	Treatment1)			SEM	p-value

	C	T1	T2		
Initial weight (kg)	711.29	712.57	712.57	14.28	1.00
Final weight (kg)	778.43	784.86	795.86	15.79	0.50
Weight gain (kg)	67.14	72.29	83.29	4.99	0.48
Average daily gain (g/d)	560	600	690	0.04	0.47

SEM, standard error of the mean; TDN, total digestible nutrient; CP, crude protein.

1) C, control (basal diet with 74% TDN and 12% CP); T1, (basal diet enriched with treatment of methionine 69% and lysine 31%; T2, (basal diet enriched with treatment of methionine 84.65% and α-tocopherol 15.35%).

**Table 2:** Carcass traits of Hanwoo steers fed with different supplementary methionine/lysine and methionine/α-tocopherol

Variable	Treatment1)			SEM	p-value

	C	T1	T2		
Yield traits2)					
Hot carcass weight (kg)	443.8	442.8	481.8	12.97	0.94
Backfat thickness (mm)	13.40	13.80	16.00	1.04	0.91
Rib eye area (cm^2^)	76.20	74.80	81.40	1.93	0.75
Yield index3)	62.30	61.90	60.40	1.30	0.89
Yield grade4)	1.60	1.60	1.60	0.79	1.00
Quality traits					
Marbling score5)	3.40	4.00	3.40	0.31	0.51
Meat color6)	3.00	3.00	4.00	0.00	0.41
Fat color7)	4.00	4.00	4.00	0.00	0.58
Firmness8)	1.00	1.40	1.80	0.09	0.24
Maturity9)	3.00	3.00	2.80	0.07	0.35
Quality grade10)	2.80	3.00	3.40	0.17	0.79

SEM, standard error of the means; TDN, total digestible nutrient; CP, crude protein.

1) C, control (basal diet with 74% TDN and 12% CP); T1, (basal diet enriched with treatment of methionine 69% and lysine 31%; T2, (basal diet enriched with treatment of methionine 84.65% and α-tocopherol 15.35%).

2) Area was measured from *longissmus thoracis* muscle.

3) Yield index was calculated by equation 65.834–[0.393×Backfat thickness (mm)]+[0.088×Ribeye area (cm^2^)]–[0.008×carcass weight (kg)]+2.01.

4) A grade (yield index ≥69.00) = 3, B grade (66.00≤yield index<69.00) = 2, C grade (yield index<66.00) = 1.

5) Marbling score standard; No.1-No.9 (1 = devoid, 9 = abundant).

6) Meat color standard; No.1-No.7 (1 = brightly cherry red, 7 = extremely dark red).

7) Fat color standard; No.1-No.7 (1 = white, 7 = dark yellow).

8) Firmness score; 1 to 3 (1 = soft, 3 = firm).

9) Maturity score; 1 to 3 (1 = youthful, 3 = mature).

10) 1++ grade = 5, 1+ grade = 4, 1 grade = 3, 2 grade = 2, 3 grade = 1.

**Table 3:** The proximate composition and physical properties of *longissimus lumborum* from Hanwoo steers fed with different supplementary methionine/lysine and methionine/α-tocopherol

Variable	Treatment1)			SEM	p-value

	C	T1	T2		
Proximate composition (%)					
Mositure	67.48	66.84	64.59	0.97	0.38
Crude fat	14.07	13.39	14.75	0.47	0.81
Crude protein	17.47^b^	18.78^a^	19.69^a^	0.71	<0.01
Crude ash	0.99	0.99	0.97	0.00	0.41
Physical properties					
Water holding capacity (%)	74.87^b^	81.31^a^	75.38^b^	0.98	<0.01
Cooking loss (%)	29.45	27.64	28.65	0.61	0.52
Shear force (kg)	6.01^a^	4.70^b^	4.90b	0.24	<0.05

SEM, standard error of the means; TDN, total digestible nutrient; CP, crude protein.

1) C, control (basal diet with 74% TDN and 12% CP); T1, (basal diet enriched with treatment of methionine 69% and lysine 31%; T2, (basal diet enriched with treatment of methionine 84.65% and α-tocopherol 15.35%).

a,b Means with different letters are different significantly from each other (p<0.05).

**Table 4:** Effect of met/lys and met/α-tocopherol supplementation on TBARS level (mg malonaldehyde/kg meat) and pH of *longissimus lumborum* from Hanwoo steers during refrigerated storage

Storage period (d)	Treatment1)			SEM	p-value		
	
	C	T1	T2		Diet	Storage	Diet×storage
TBARS (mg malonaldehyde/kg meat)							
0	0.32^a^	0.32^a^	0.28^b^	0.019	<0.05	<0.001	0.3
3	0.43^a^	0.4^a^	0.35^b^	0.008			
6	0.49^a^	0.43^ab^	0.39^b^	0.028			
9	0.6^a^	0.45^b^	0.39^c^	0.033			
pH value							
0	5.4^a^	5.4^a^	5.35^b^	0.010	<0.05	<0.001	0.31
3	5.4	5.44	5.4	0.014			
6	5.43^ab^	5.46^a^	5.4^b^	0.009			
9	5.61	5.55	5.49	0.054			

TBARS, 2-thiobarbituric acid reactive substances; SEM, standard error of the means; TDN, total digestible nutrient; CP, crude protein.

1) C, control (basal diet with 74% TDN and 12% CP); T1, (basal diet enriched with treatment of methionine 69% and lysine 31%; T2, (basal diet enriched with treatment of methionine 84.65% and α-tocopherol 15.35%).

a–c Means with different letters are different significantly from each other (p<0.05).

**Table 5:** Effect of met/lys and met/α-tocopherol supplementation on meat color and relative myoglobin concentration (%) of *longissimus lumborum* from Hanwoo steers during refrigerated storagemet/lysmet/α-tocopherol

Items	Storage (d)	Treatment1)			SEM	p-value		
	
		C	T1	T2		Diet	Storage	Diet×storage
Meat color								
L*	0	38.8	37.73	38.89	1.36	<0.05	0.82	0.73
	3	38.22	37.26	39.88	0.38			
	6	37.6	35.55	38.13	0.99			
	9	38.02^ab^	37.12^b^	39.54^a^	0.43			
a*	0	24.9	24.66	25.89	1.36	<0.001	<0.001	0.31
	3	23.31^b^	23.24^b^	25.35^a^	0.38			
	6	22.68^b^	22.71^b^	24.6^a^	0.99			
	9	16.8^b^	17.55^b^	20.96^a^	0.43			
b*	0	13.83	13.83	13.75	0.27	0.5	<0.001	0.9
	3	13.69	12.27	13.18	0.32			
	6	12.13	12.24	12.73	0.10			
	9	9.09	10.56	11.06	0.42			
Myoglobin profile								
Oxymyoglobin (%)	0	57.4	60.5	61.3	1.98	<0.05	<0.001	0.74
	3	45.8	46.9	50.8	0.90			
	6	32.6^b^	38.6^a^	39.1^a^	1.70			
	9	15.3^b^	23.3^a^	26.3^a^	1.15			
Metmyoglobin (%)	0	22.96	21.96	20.51	1.09	0.11	<0.001	0.91
	3	34.71	34.85	33.11	1.00			
	6	38.48	35.82	34.17	0.57			
	9	52.61	50.53	47.86	0.83			
Deoxymyoglobin (%)	0	19.66	17.58	18.21	1.36	0.16	<0.01	0.54
	3	19.54	18.69	16.67	0.38			
	6	28.91	26.03	25.45	0.99			
	9	32.07	27.6	27.27	0.43			

SEM, standard error of the means; TDN, total digestible nutrient; CP, crude protein.

1) C, control (basal diet with 74% TDN and 12% CP); T1, (basal diet enriched with treatment of methionine 69% and lysine 31%; T2, (basal diet enriched with treatment of methionine 84.65% and α-tocopherol 15.35%).

a,b Means with different letters are different significantly from each other (p<0.05).

**Table 6:** Effect of met/lys and met/α-tocopherol supplementation on total fatty acid concentration (%) of Hanwoo steers

Items	Treatment1)			SEM	p-value

	C	T1	T2		
C14:0	3.05^b^	3.74^a^	3.37^ab^	0.14	<0.05
C16:0	28.55	29.13	27.91	0.42	0.53
C16:1n7	5.62	5.59	5.74	0.18	0.95
C18:0	9.62	9.78	9.97	0.34	0.93
C18:1n9	50.50	49.21	50.19	0.60	0.67
C18:2n6	2.37	2.28	2.45	0.08	0.59
C18:3n3	0.24	0.21	0.27	0.02	0.40
C20:4n6	0.05	0.04	0.06	0.01	0.30
C20:5n3	0.04	0.03	0.02	0.00	0.14
SFA	41.22	42.65	41.24	0.63	0.61
MUFA	56.12	54.79	55.92	0.64	0.69
PUFA	2.66	2.56	2.83	0.09	0.48
n-3	0.28	0.24	0.29	0.02	0.49
n-6	2.38	2.32	2.54	0.08	0.56
n-6/n-3	9.00	10.10	8.71	0.55	0.59

SEM, standard error of the means; TDN, total digestible nutrient; CP, crude protein; SFA, saturated fatty acid; MUFA, mono unsaturated fatty acid; PUFA, poly unsaturated fatty acid.

1) C, control (basal diet with 74% TDN and 12% CP); T1, (basal diet enriched with treatment of methionine 69% and lysine 31%; T2, (basal diet enriched with treatment of methionine 84.65% and α-tocopherol 15.35%).

a,b Means with different letters are different significantly from each other (p<0.05).
